# Local electronic structure of doping defects on Tl/Si(111)1x1

**DOI:** 10.1038/s41598-018-37361-5

**Published:** 2019-01-28

**Authors:** Barbara Pieczyrak, Leszek Jurczyszyn, Pavel Sobotík, Ivan Ošt’ádal, Pavel Kocán

**Affiliations:** 1 Instytut Fizyki Doswiadczalnej, Universytet Wroclawski, pl. Maksa Borna 9, 50-204 Wroclaw, Poland; 2 Charles University, Faculty of Mathematics and Physics, Department of Surface and Plasma Science, V Holešovičkách 2, 180 00, Prague, Czech Republic

## Abstract

The Tl/Si(111)1 × 1 surface is a representative of a 2D layer with Rashba-type spin-split electronic bands. To utilize the spin polarization, doping of the system should be understood on atomic level. We present a study of two types of atomic defects predicted to dope the considered electronic system – Si-induced vacancies and defects associated with the presence of extra Tl atoms. Structural calculations based on density functional theory (DFT) confirm the stability of the proposed defect structure consisting of an extra Si atom and missing seven Tl atoms as proposed in an earlier experimental study. The calculated spatial charge distributions indicate an enhancement of the charge around the extra Si atom, which correctly reproduces topographies of the corresponding scanning tunneling microscopy images while the calculated local densities of states of this system explain obtained scanning tunneling spectra. The DFT structural calculations let us determine the atomic structure of the defect caused by the presence of an extra Tl atom. The calculated spatial charge distributions show a ring-like feature around the extra Tl atom. The obtained results indicate a charge transfer from the central extra Tl atom to its vicinity in the agreement with earlier photoemission measurements.

## Introduction

Two-dimensional (2D) systems composed of densely packed monolayer of metal adatoms on Si(111) or Ge(111) surface show fascinating electronic properties. For example, nearly massless electrons^1^ and 2D superconductivity^2^ have been reported in the case of Pb monolayer on the Si(111) surface. Other related systems involving heavy elements, such as Tl^3–10^ and Bi^11,12^ on the Si(111), or Au^13^ and Pb^14^ on the Ge(111) show strongly spin-split surface-state bands, the splitting being caused by the Rashba effect^15,16^.

Electronic structures of Bi/Si(111) and Tl/Si(111) have been modified by the formation of 2D alloys with other elements^17,18^, or by sandwiching a Sn monolayer between Si(111) and the heavy element layer^19^. This resulted in metallic spin-split bands instead of the otherwise semiconducting bands formed by Bi or Tl on Si(111). In the case of a (Tl, Pb) alloy on Si(111), superconductivity and Rashba splitting have both been combined^18^.

In order to bring these exotic properties into working devices, tuning of the electronic structure, i.e. doping, needs to be possible. Chemical doping of graphene–a prototype of a 2D electron system heading to practical applications^20^–is possible and is being extensively studied (see e.g. ref.^21^ and references therein). So far, similar studies for 2D metal monolayer systems are much more rare^22,23^.

The monolayer Tl/Si(111)1×1 system has a pseudomorphic 1×1 structure with each Tl atom in a T_4_ position above the as-bulk terminated Si(111) surface^24–26^. Electronically the layer exhibits a giant spin-orbit-induced spin splitting of states in the bulk band gap^3–10^. The complex spin texture of the spin-polarized states has been studied in detail^5,8–10^ and the spin-split bands have been shown to be robust against exposure to H_2_ (100 L) and O_2_ (500 L)^10^.

Upon further deposition of Tl, isolated defects were observed at low temperature and low extra Tl amounts^6^, while the second layer with a 6 × 6 moiré pattern is formed at room and higher temperatures and at higher deposited amounts^27,28^. Notably, the double layer shows superconducting transition at 0.96 K^29^. A structural model of the double-layer has been proposed recently containing 1.2 monolayer (ML, 1 ML = 7.83 × 10^14^ atoms cm^−2^) of Tl atoms in each layer^30^.

Two previous works are important for this study. First, we proposed a structural model of the ring-shaped defects observed in scanning tunneling microscopy (STM), consisting of a single extra Si atom inducing a Tl multivacancy^31^. Furthermore, we demonstrated the metallicity of the observed layers in contrast to predictions from density functional theory (DFT) calculations and discussed a possible doping effect of the defects. The defects also play an important role in the interaction with other adsorbates on the surface^32,33^. In the second important work, Sakamoto *et al*. reported that the unoccupied spin-split band of Tl/Si(111)1 × 1 can be shifted towards the Fermi level by the deposition of extra Tl atoms^6^. The extra Tl atoms therefore represent n-type dopants to the layer. This rigid band-shifting model has been recently debated^10^ as angle-resolved photoemission (ARPES) and inverse-photoemission experiments have shown that the states distant from the Fermi level did not shift upon extra Tl deposition. Instead, a new state at the Fermi level appeared. However, the experimental conditions used in ref.^10^ resulted rather in the formation of a local double-layer instead of randomly separated atomic defects as in ref.^6^. This could explain the discrepancy as a doping shift of states would be observed only in the latter case.

Here we present DFT calculations of Tl structures with doping defects–Tl adatoms and Si-induced vacancies–and discuss their influence on the electronic structure together with the related redistribution of states in the real space.

## Results and discussion

### Si-induced multivacancies

**Figure 1:**
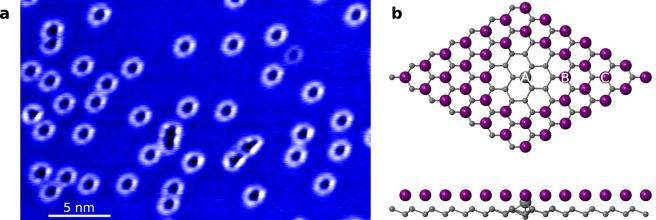
(**a**) STM image of the Tl/Si(111)1×1 surface at sample voltage *U*_*S*_ = +1 V showing ring-shaped defects. (**b**) Top and side views of the relaxed structure of the multi-vacancy defect on the Tl/Si(111)1×1 surface induced by the presence of an extra Si atom at the topmost atomic layer and missing Tl atoms. Only topmost Si bi-layer of the slab used in the calculations is shown. The Tl adatoms are marked by large violet spheres, the extra Si atom by a large gray sphere and the Si substrate atoms by small gray spheres.

Figure 1a shows a representative STM image of the Tl/Si(111)1×1 surface with ring-shaped defects. Previously we proposed a structural model, in which seven Tl atoms of the 1×1 structure are replaced by a single Si ad-atom in a T_4_ position^31^ (see Fig. 1b). These extra Si ad-atoms could easily originate from the Si-rich 7 × 7 reconstruction present on the surface prior to the formation of the Tl/Si(111)1×1 structure. The model is also consistent with the tendency of Si ad-atoms to substitute Tl during the formation of the mosaic $\sqrt{3}\times \sqrt{3}$ structure at elevated temperatures^34,35^: the separation of neighboring Si and Tl ad-atoms in the $\sqrt{3}\times \sqrt{3}$ is the same as in the proposed model of the ring-shaped defects. The surface density of defects is very sensitive to the preparation parameters, primarily to the smaple heating temperature. This is probably caused by a narrow temperature interval between formation of the 1×1 structure and Tl desorption^34^. We note that other less frequent types of defects coexisting with the ring-shaped ones can be observed occasionally^32^.

**Figure 2:**
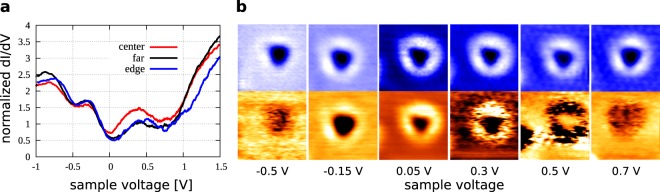
(**a**) STS spectra obtained for different positions of the STM tip with respect to the defect atomic structure. (**b**) Constant current STM images (top) and constant-height d*I*/d*V* maps of the defect (bottom). Sample voltages are indicated.

In order to study the local electronic structure of the defects, we measured scanning tunneling spectroscopy (STS) curves above the center of the defect, above the edge of the defect, as well as above a Tl atom far from the defect (Fig. 2a). The most significant feature of the STS obtained above the center is enhanced density of empty states up to sample voltage 0.6 V. The enhancement is balanced out by a lower density of occupied states above the defect center compared to the edge and the faraway position. The positive charging of the central Si atom and consequent hole doping is consistent with the mechanism proposed previously^31^: Si dangling bonds of the pristine Tl/Si(111)1×1 structure are saturated by a charge transfer from Tl *p* orbitals. The formation of a Tl multi-vacancy prevents some of the Si dangling bonds to be saturated via Tl atoms. The charge from the extra Si atom is therefore transferred to participate in the saturation of these Si dangling bonds.

Figure 2b shows detailed bias-dependent constant current STM images (top panels) and d*I*/d*V* maps (bottom panels) of a selected defect. The somewhat lower quality of the images is caused by the simultaneous recording of the current (used as feedback in the topography images shown in the top panels) and the lock-in d*I*/d*V* signal (imaged with feedback switched off shown in the bottom panels). The STM topography shows just a minor dependence on the sample voltage except a change of the relative contrast of the central and Tl-1×1 areas. On the other hand, the d*I*/d*V* maps show a stronger dependence - the ring of the defect appears brighter from −0.15 V to 0.3 V, the central position appears brighter at 0.5 V, otherwise the defect is displayed as a depression.

**Figure 3:**
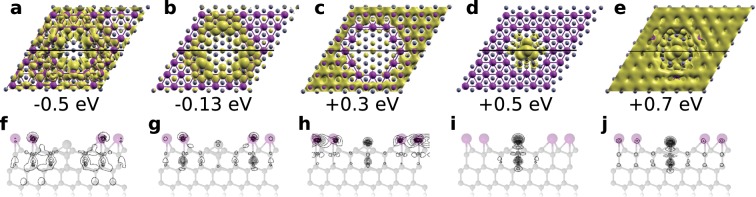
Spatial charge distributions (**a**–**e**) of electronic states at indicated energies. (**f**–**j**) Calculated profiles along the black lines indicated in (**a**–**e**). Used isovalues are (**a**) 0.005 e/Å^3^, (**b**,**c**) 0.007 e/Å^3^, (**d**–**e**) 0.001 e/Å^3^.

To verify the proposed model of the defect we have calculated the spatial charge distributions of the electronic states contributing to the STM images obtained from the measurements (Figs 1a and 2b). Figure 3 shows charge distributions of the filled (Fig. 3a,b) and empty (Fig. 3c–e) states near the Fermi level obtained from calculations including spin-orbit coupling.

**Figure 4:**
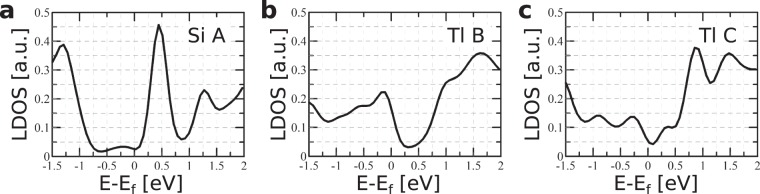
LDOS projected onto the orbitals of extra Si atom located in the center of the defect (**a**), Tl atom from the ring around extra Si atom (**b**), and Tl atom located far from the defect - (**c**). Denotation of particular atoms is the same as in Fig. 1b.

Charge distributions of the modeled defect shown in Fig. 3 reproduce the enhancement in the ring around the extra Si atom indicated by the STM images (see Figs 1a and 2b). The ring decoration is strongest at −0.13 eV (Fig. 3b,g) which corresponds to the d*I*/d*V* map obtained at −0.15 eV in Fig. 2b. The local density of states (LDOS) associated with the ring is significant even at the Fermi level (see Fig. 4b) and consequently this type of defect appears as a bright circle in STM images at a wide range of sample voltages. The density of states inside the ring is maximal at 0.5 eV (see Fig. 3d). The charge distribution associated with the extra Si atom is visible under some conditions of STM tips^31^. At 0.3 eV the increased charge density can be found in Fig. 3h, but it does not protrude out of the Tl layer. The protrusion is not visible even in the the d*I*/d*V* map at 0.3 eV (Fig. 2b). However, the increased LDOS can be identified in the STS curve (red line in Fig. 2a) as the enhancement between the Fermi level and 0.7 eV. Such different sensitivity to the charge density located at the central atom is due to different tip setpoints: the tip is closer to the surface during the STS measurement as tip height is controlled by the feedback loop during STM image acquisition in contrast to the constant height during the d*I*/d*V* mapping.

The results obtained from the structural calculations indicate that the vertical positions of the Tl atoms forming the ring around the extra Si atom are slightly, around 0.04 Å, lowered with respect to the corresponding values obtained for Tl atoms located far from the defect. Their lateral positions are shifted toward the extra Si atom by 0.08–0.1 Å. The vertical position of the extra Si atom in the centre of the defected area is much lower, by around 1.17 Å, in comparison with Tl atoms of the pristine Tl/Si(111)1×1 structure.

To compare the electronic structure of the defect with the results of STS measurements, we performed calculations of the LDOS associated with the different atoms of the defected area. Figure 4 shows the LDOS projected onto the orbitals of the extra Si atom situated in the centre of the ring (Fig. 4a), the selected Tl atom from the ring in Fig. 4(b) and the Tl atom located far from the defect (Fig. 4c).

The calculated LDOS shown in Fig. 4 can explain some features of the corresponding experimental data provided by STS measurements. The STS spectra presented in Fig. 2a indicate the feature located around 0.4 eV above the Fermi level. This feature is the most pronounced when the tip is located above the central extra Si atom. The corresponding maximum in the LDOS projected onto the orbitals of the extra Si atom (Fig. 4a) is located 0.4–0.5 eV above the Fermi level. The localization of electronic states associated with this LDOS feature is illustrated by the corresponding spatial charge distribution shown in Fig. 3d,i. The shape of the LDOS calculated for the Tl atoms from the ring around the extra Si atom (Fig. 4b) shows an enhancement of occupied states with a maximum located at −0.13 eV and a non-zero density at the Fermi level. The corresponding spatial distribution is shown in Fig. 3b,g.

We have noticed that the electronic properties of the defected area obtained from our theoretical study with spin-orbit coupling correctly reproduce the corresponding experimental STM data which gives strong support for the model of the defect with an extra Si atom proposed previously^31^.

### Extra Tl atoms

In the next part of our study we consider the Tl/Si(111)1×1 surface modified by additional Tl adatoms. Such a surface system has been studied experimentally by Sakamoto *et al*.^6^. Their ARPES results indicate that a very small amount of extra Tl adatoms (extra Tl coverage of 0.01 ML) shifts the originally unoccupied band of electronic states to the Fermi level. With increasing coverage of extra Tl adatoms this band continuously moves towards lower energies.

**Figure 5:**
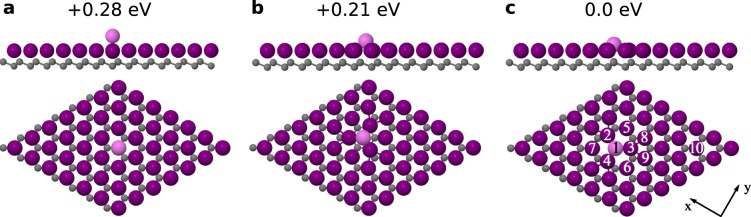
Side and top views of the relaxed atomic structures of the Tl/Si(111)1×1 surface modified by the presence of an extra Tl atom (coverage of 0.02 ML). Only the topmost Si bi-layer of the slab used in the calculations is shown. The Tl adatoms are marked by large violet spheres, the extra Tl atom by a large pink sphere and the Si substrate atoms by small gray spheres. (**a**–**c**) Represent the structures with extra Tl atom adsorbed in on-top position, shallow hollow site and deep hollow site, respectively. The relative energies with respect to the energetically most stable configurations (deep hollow site) and the orientation of the x-y plane are also given.

We analyzed the geometrical and electronic properties of the Tl/Si(111)1×1 surface system containing extra Tl adatoms with structural ab-initio calculations. We used a 7 × 7 surface unit cell with an extra Tl adatom (coresponding coverage 1.02 ML). Relaxation has been performed for the three following positions of the extra Tl adatom: on-top (with respect to Tl adatoms from the preadsorbed adlayer), shallow hollow site and deep hollow site. All three configurations after structure relaxation are shown in Fig. 5. The total energy calculation results indicate that the deep hollow site configuration (Fig. 5c) represents the lowest energy configuration: the shallow hollow and on-top have higher energies by 0.21 eV and 0.28 eV, respectively. Please note the tendency of the extra Tl atom to incorporate into the Tl monolayer (Fig. 5c), which is in agreement with a proposed structural model of the double layer with both layers having a density of 1.2 ML^30^.

**Figure 6:**
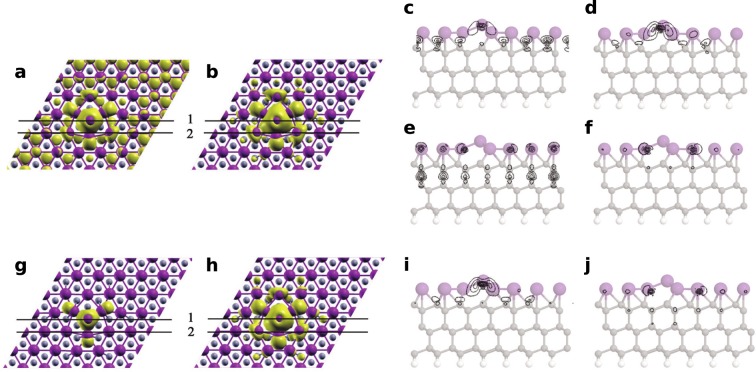
Spatial charge distributions calculated for the adsorption system with an extra Tl atom in the deep hollow configuration. (**a**) Represents the occupied electronic states in the energy range between *E*_*f*_ − 0.1 eV and *E*_*f*_, while (**b**) corresponds to the unoccupied states in the range between *E*_*f*_ and *E*_*f*_ + 0.1 eV. The isosurface values are 0.001 e/Å^3^ and 0.0003 e/Å^3^, respectively. (**c**,**d**) Calculated profiles along the black lines labeled 1 in (**a**,**b**); (**e**,**f**) Calculated profiles along the black lines labeled 2 in (**a**,**b**). (**g**,**h**) are charge distributions for the electronic states in the energy range from *E*_*f*_ up to 0.2 eV above *E*_*f*_ with isosurface value 0.008 e/Å^3^ and 0.0004 e/Å^3^. (**i**,**j**) corresponding calculated profiles along the black lines indicated in (**g**,**h**), (**i**) corresponds to line 1 and (**j**) to line 2.

To compare the electronic properties of the system with STM data^6^, we have calculated the partial charge distributions. The obtained results are presented in Fig. 6a,b and in the profiles in Fig. 6c–f–these distributions illustrate the localization of the electronic states in the vicinity of the Fermi level (*E*_*f*_). Figure 6a presents the partial charge distribution for the occupied states in the energy range from *E*_*f*_ − 0.1 eV to *E*_*f*_, while Fig. 6b represents the corresponding distribution obtained for the unoccupied states in the range from *E*_*f*_ to *E*_*f*_ + 0.1 eV. We have obtained a qualitative agreement between the distribution calculated for the unoccupied states (Fig. 6b) and the topography of the corresponding STM image^6^. However, the interpretation of STM images of extra Tl atoms from ref.^6^ is complicated by the fact that the reported shape and size may be affected by a complex electron interference at the defects. The apparent size of the extra Tl atoms^6^ is ∼1.3 nm, which is larger than the partial charge distribution shown in Fig. 6a,b. In a similar way, atomic-scale defects e.g. in the graphene lattice appear in STM topographic images as larger objects with a regular shape^36^. Furthermore, we shall discuss in detail the calculated local densities of states related to the presence of the extra Tl atom with the structure presented in Fig. 5c.

**Figure 7:**
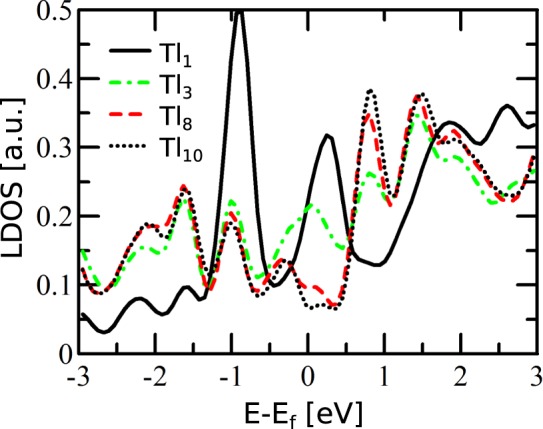
DOS distributions projected onto the orbitals of selected topmost Tl atoms of the system shown in Fig. 5c. Denotation of the particular atoms is the same as in Fig. 5c.

Figure 7 shows the DOS distributions projected onto orbitals of selected topmost atoms of the considered system. The labeling of the atoms used hereafter is shown in Fig. 5c. The DOS distribution associated with the atom Tl_10_, which is located far from the extra Tl adatom (Tl_1_), shows a sharp maximum located 0.8 eV above the Fermi level (black dotted curve in Fig. 7). This result corresponds well with the presence of the unoccupied spin-down band indicated by the SARIPES measurements^6^ performed for the system without the extra Tl atom. On the other hand, the small DOS feature related to the Tl_10_ atom located just above the Fermi level can be linked with the spin-up band indicated by the corresponding SARIPES measurements^6^. In the case of the atom Tl_3_, which is situated in the nearest neighborhood of the extra Tl atom (Tl_1_), the additional DOS maximum appears at the Fermi level (dotted-dashed green curve in Fig. 7). The presence of such partially filled states is associated with a charge transfer from the extra Tl atom to its vicinity.

**Figure 8:**
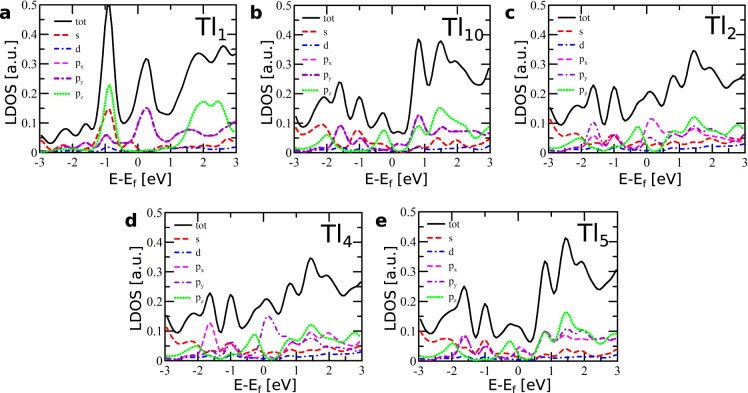
DOS distributions projected onto the orbitals of selected atoms: (**a**) extra Tl atom, (**b**) Tl atom located far from the defect, (**c**,**d**) nearest neighbors of extra Tl atom (Tl_3_ is equivalent to Tl_2_), (**e**) second neighbors of extra Tl atom (Tl_6_ and Tl_7_ are equivalent to Tl_5_). Denotation of the atoms used for DOS calculation is the same as in Fig. 5c.

In STM empty states images^6^ and also in spatial charge distributions, the extra-Tl-defect is represented by a ring-like feature with a hollow in its center. The origin of the central hollow can be rationalized with the analysis of the DOS distributions associated with the extra Tl atom (atom Tl_1_ - black solid curve in Fig. 7). This distribution shows a sharp maximum located just above the Fermi level, but the analysis presented in Fig. 8a indicates that this DOS feature is composed almost solely of *p*_*x*_ − *p*_*y*_ orbitals which are oriented parallel to the surface. For comparison, Fig. 8b shows the corresponding dependences for Tl atom located far from the defect (atom Tl_10_ in Fig. 5c). The dominance of the *p*_*x*_ − *p*_*y*_ states of the extra Tl atom near the Fermi level is responsible for imaging the atom as a hollow in STM–these states are visible not above the extra-Tl atom but around it, as it is shown in Fig. 6. The DOS distribution presented in Fig. 8a shows that this argument can be also extended to the occupied states located just below the Fermi level.

The ring-like feature in the partial charge distribution (Fig. 6a,b), which appears around the central extra Tl atom, represents the result of an interaction between *p*_*x*_ − *p*_*y*_ states of the extra Tl atom (atom Tl_1_ in Fig. 5c) and its three nearest neighbors - atoms Tl_2_, Tl_3_ and Tl_4_. Figure 8c and d present the DOS distributions projected onto orbitals of the atoms Tl_2_ and Tl_4_–the corresponding DOS distribution and its components related to the Tl_3_ atom is the same as for the Tl_2_ atom and therefore not shown in the figure. The obtained results indicate the presence of distinct DOS features located just above the Fermi level and composed of *p*_*x*_ − *p*_*y*_ states associated with the Tl_2_, Tl_3_ and Tl_4_ atoms. The interaction of these states with the *p*_*x*_ − *p*_*y*_ orbitals of the extra Tl atom leads to the formation of inter-orbital hybrid shown in Fig. 6g which presents the same spatial charge distributions as shown in Fig. 6h but a greater isovalue has been used this time–and therefore only the contributions associated with the Tl_2_, Tl_3_ and Tl_4_ (and also Tl_1_) atoms are visible.

The contributions to the ring-like feature around the extra Tl atom come also from the three second nearest neighbors (with respect to the extra Tl atom)–atoms Tl_5_, Tl_6_ and Tl_7_ in Fig. 5c. Figure 8e presents the DOS distribution (together with its components) associated with the Tl_5_ atom - the corresponding distributions related to the Tl_6_ and Tl_7_ atoms are exactly the same and are therefore are not shown in Fig. 8. These distributions show a maximum located just above the Fermi level and composed solely of *p*_*x*_ − *p*_*y*_ states. These DOS features are much smaller than the *p*_*x*_ − *p*_*y*_ DOS maxima located in the same energy range stemming from the first neighbors (i.e. atoms Tl_2_, Tl_3_ and Tl_4_), but are considerably higher than analogous contributions for a Tl atom located far from the defect (atom Tl_10_ in Fig. 5c).

The contribution associated with the *p*_*x*_ − *p*_*y*_ states of Tl_5_, Tl_6_ and Tl_7_ atoms to the formation of the ring-like feature surrounding the extra Tl atom in spatial charge distributions is confirmed by the corresponding distribution presented in Fig. 6h. In comparison with Fig. 6g, the isovalue applied in Fig. 6h is reduced, and therefore the features associated with the first neighbors (atoms Tl_2_, Tl_3_, Tl_4_) and also second neighbors (atoms Tl_5_, Tl_6_, Tl_7_) are visible. It also follows from Fig. 6h that the *p*_*x*_ − *p*_*y*_ orbitals of the second neighbors interact with the *p*_*z*_ states of the two nearest silicon atoms - this inter-orbital hybrid is also influenced by the contact with the *p*_*x*_ − *p*_*y*_ states associated with the extra Tl atom (atom Tl_1_).

It is worth mentioning that the features associated with the *p*_*x*_ − *p*_*y*_ states of the Tl atoms located far from the defect are not visible in the calculated spatial charge distributions since the *p*_*x*_ − *p*_*y*_ contributions to the density of states of these Tl atoms are much smaller in the vicinity of the Fermi level than those of the TL atoms closest to the defect.

Our theoretical study clearly shows that extra Tl atoms modify the electronic properties of Tl/Si(111) by doping the Tl layer. In our view, this result is in agreement with the experimental data^6^.

## Conclusions

We presented a combined theoretical and experimental study of the Tl/Si(111) adsorption system with Si-induced vacancies and also defects implied by the presence of extra Tl adatoms.

We considered a Tl/Si(111) system defected by an extra Si atom and missing seven Tl atoms, structure of which is based on a model proposed in the earlier STM study^31^. To verify the stability of this model structure and to check its agreement with existing STM data, structural DFT calculations were performed. The obtained results indicate that the assumed model represents a stable structure and the calculated electronic properties of the relaxed defected system can explain some important features of the experimental STM/STS data. We found that the spatial charge distribution of these electronic states, which are active during the formation of STM images, reproduces the characteristic ring-shape feature visible around the extra Si atom in STM images. We also showed that the LDOS distributions projected onto the orbitals of the extra Si atom located in the centre of the defect, a Tl atom from the ring around the extra Si atom and a Tl atom located far from the defect, reproduce the corresponding experimental data obtained from STS measurements.

The theoretical study of the Tl/Si(111) system defected by the presence of the extra Tl atom allowed us to determine its energetically optimal structure. The presented calculated spatial charge distributions show that the STM images obtained earlier by Sakamoto *et al*. might be related to the ring-like contour associated with the three nearest Tl atoms with respect to the extra Tl atom and its three second nearest neighbors. The calculated LDOS distributions indicate a charge transfer from the extra Tl atom to its nearest surrounding. The charge transfer is important for doping of the Tl/Si(111)1×1 surface.

## Methods

### DFT

The theoretical studies were performed with the use of periodic slab ab-initio calculations based on the Density Functional Theory (DFT) as implemented in the Vienna Ab-initio Simulation Package (VASP)^37–40^. The electron-ion interaction was described by the projector-augmented wave (PAW) potential^41,42^ where the following angular momentum channels have been assumed: Si(s,p,d) and Tl(s,p,d). To include the exchange-correlation contribution the generalized gradient approximation (GGA) in its PBE formulation was used^43^. These calculations had fully relativistic non-collinear character–the spin-orbit interaction was included. All calculations were performed at 0K, and thus the obtained structural model and the atomic structure at finite temperature can be different.

The description of the substrate was performed with the use of an asymmetric slab consisting of six silicon layers constructed using the calculated bulk lattice constant 5.437 Å. The dangling bonds at the bottom of the slab were saturated by hydrogen atoms. The Tl ad-layer was modeled by positioning the Tl ad-atoms in the T_4_ adsorption site of the Si(111)1×1 substrate. During the relaxation the atomic positions inside the ad-layer and the four topmost silicon layers were allowed to relax until the forces were less than 0.01 eV/Å while the rest part of the slab was frozen in its bulk-like configuration. All results presented in this paper were obtained for one *k*-point (Γ point), however, the influence of this factor was tested in separate check calculations. The applied cutoff energy in all calculations presented in this study was 450 eV. The calculated energy gap of the ideal Tl/Si(111)1×1 equals 0.1 eV. To analyze the structural and electronic properties of the defects (the ring-shaped defect described previously in ref.^31^ and the extra Tl adatom defect) we considered 7 × 7 unit cells.

### STM

The measurements were performed at room temperature in a noncommercial ultra-high-vacuum STM system. The pressure in the system did not exceed 1×10^−8^ Pa during experiments. The Si(111) samples (Sb doped, with a resistivity of 0.005–0.01 Ω cm) were cleaned by flashing to 1200  C. Thallium (purity 99.999%) was evaporated onto the silicon surface at room temperature, with the amount controlled by a quartz thickness monitor. The Tl/Si(111)1×1 surface was prepared by heating the sample, predeposited with Tl amount exceeding one ML, to 300  C for 2 min. The sample was resistively heated by passing dc current, and the sample temperature was determined from calibrated heating power. STM tips were prepared by electrochemical etching from a polycrystalline tungsten wire and treated *in situ* by an electron bombardment^44^. The d*I*/d*V* curves were measured using a lock-in technique (∼10 s per spectrum), and the presented data were averaged over several equivalent spectra normalized by *I*/*V*. Noise near the Fermi level was suppressed by a procedure from ref.^45^. Lock-in d*I*/d*V* maps were acquired in constant height mode with STM feedback loop switched off.

## Data Availability

The datasets generated during and/or analysed during the current study are available from the corresponding author on reasonable request.
